# High-performance hydrogen gas sensor system based on transparent coaxial cylinder capacitive electrodes and a volumetric analysis technique

**DOI:** 10.1038/s41598-024-52168-3

**Published:** 2024-01-23

**Authors:** Jae K. Jung, Ji H. Lee

**Affiliations:** 1 Hydrogen Energy Materials Research Team, Korea Research Institute of Standards and Science, Daejeon, 34113 Korea; 2 Department of Measurement Science, University of Science and Technology, 217 Gajeong-ro, Yuseong-gu, Deajeon, 34113 Korea

**Keywords:** Electronics, photonics and device physics, Techniques and instrumentation, Energy science and technology, Physics

## Abstract

A high-performance H_2_ gas sensor system based on capacitive electrodes and a volumetric analysis technique were developed. Coaxial capacitive electrodes were fabricated by placing a thin copper rod in the center and by adhering a transparent conductive film on the exterior surface of a graduated cylinder. Thus, H_2_ from a polymer specimen lowered the water level in the cylinder between the two electrodes, producing measurable changes in capacitance that allowed for the measurement of the H_2_ concentration emitted from the specimen enriched by H_2_ under high-pressure conditions. The sensing system detected diffused/permeated hydrogen gas from a specimen and hydrogen gas leaks caused by imperfect sealing. The hydrogen gas sensor responded almost instantly at 1 s and measured hydrogen concentrations ranging from 0.15 to 1500 ppm with controllable sensitivity and a measurable range. In addition, performance tests with polymer specimens used in hydrogen infrastructure verified that the sensor system was reliable; additionally, it had a broad measurement range to four decimal places. The sensor system developed in this study could be applied to detect and characterize pure gases (He, N_2_, O_2_ and Ar) by real time measurement.

## Introduction

Hydrogen, which is the ultimate energy source, is exponentially increasing in attention throughout the global energy market due to great prospects for replacing conventional fossil energy sources and reducing harmful CO_x_/NO_x_ emissions^1–6^. Despite being a clean and sustainable source of energy, the explosive and flammable nature of hydrogen must be considered critically in every process, especially when the hydrogen content ranges from 4 to 75% in air^7,8^. Therefore, the detection of low concentrations of H_2_ is essential for reducing the danger of explosions caused by the leakage of H_2_ during production, transportation, storage and use in stationary and mobile applications.

Polymers are widely used as seals in hydrogen infrastructure systems that are exposed to high-pressure (HP) H_2_ during service^9–12^. O-ring sealings, control valves, gaskets, liner materials and non-metallic pipelines are the major application of polymeric materials^13–17^. In harsh environments with wide temperature changes (− 50 ~ 90 °C) and numerous pressure cycles (0 ~ 90 MPa), H_2_ leaks can form in different situations, such as seal damage, insufficient contact between seals and equipment, and gas permeation through polymeric seals. Therefore, real-time sensing of hydrogen leaks and in situ measurements of hydrogen concentrations released in the environment are constantly required for hydrogen infrastructure systems and O-ring materials.

Hydrogen gas sensors utilize diverse technologies in which a specific properties of a sensing element changes in the presence of hydrogen gas^18–26^. The property could correspond to thermal conductivity, a work function, catalytic activity, an acoustic detection method, and an optical, mechanical, chemical, or physical property. The sensor system requires a transducer to convert the specific change to an electric signal that can be further processed and analyzed. The advantages and disadvantages of each sensor type have been discussed in other studies^20^.

In addition, H_2_ detection methods utilize specific instruments, such as gas chromatograph, mass spectrometer and ionization gas pressure sensor. Gas chromatograph uses columns to separate the gas components of a mixture and different types of detectors and to identify each component. Mass spectrometer identifies gas molecules based on their characteristic deflections by a magnetic field. However, these instruments are relatively large and expensive, require periodic maintenance and have slow sampling processes. Moreover, H_2_ sensors should satisfy multiple requirements: rapid response, high sensitivity, good selectivity and wide measurable ranges^27–30^.

Measurements of effective, high-performance and real time are necessary to overcome the limitations of instrumental methods and enhance the performance of hydrogen sensing. In this work, we propose a H_2_ sensor system based on a volumetric technique and a combination of a coaxial capacitive electrode and frequency response analyzer (FRA) with a general purpose interface bus (GPIB) interfaced with a personal computer (PC) and diffusion/permeation analysis program. The sensor system developed in this study is effective for measuring hydrogen content, leaks and diffusion. The developed sensor allows us to solve several challenges related to the rapid, temperature/pressure-insensitive, and reliable detection of H_2_ in the field.

The two techniques developed in this study utilize two different hydrogen HP exposure conditions. One technique is the measurement of hydrogen emissions from specimens by a volumetric method after exposure to HP in a vessel and after subsequent decompression. The other technique is the measurement of permeated hydrogen from specimens by a combined volumetric and differential pressure method with a permeation cell under HP conditions.

Two methods are commonly applied for leakage or permeation tests of polymeric materials, such as nitrile butadiene rubber (NBR), ethylene propylene diene monomer (EPDM), high-density polyethylene (HDPE) and low-density polyethylene (LDPE). These polymeric materials are utilized for gas seals, such as the O-ring and liner material of compressed hydrogen pressure vessels under HP conditions^14,31^. In this study, we demonstrate the various performance results obtained by this sensor system. For instance, the dual-mode detection of H_2_ via sorption and diffusion is demonstrated for a polymer membrane. The sensor system is validated by comparison with different methods. Moreover, the sensor system developed in this study can be utilized to detect and characterize other pure gases (He, N_2_, O_2_ and Ar) through the real time measurement of pure gases in a polymer. In the conclusion, the feature of the developed gas sensor are reviewed.

## Analysis program for obtaining hydrogen permeation

**Figure 1:**
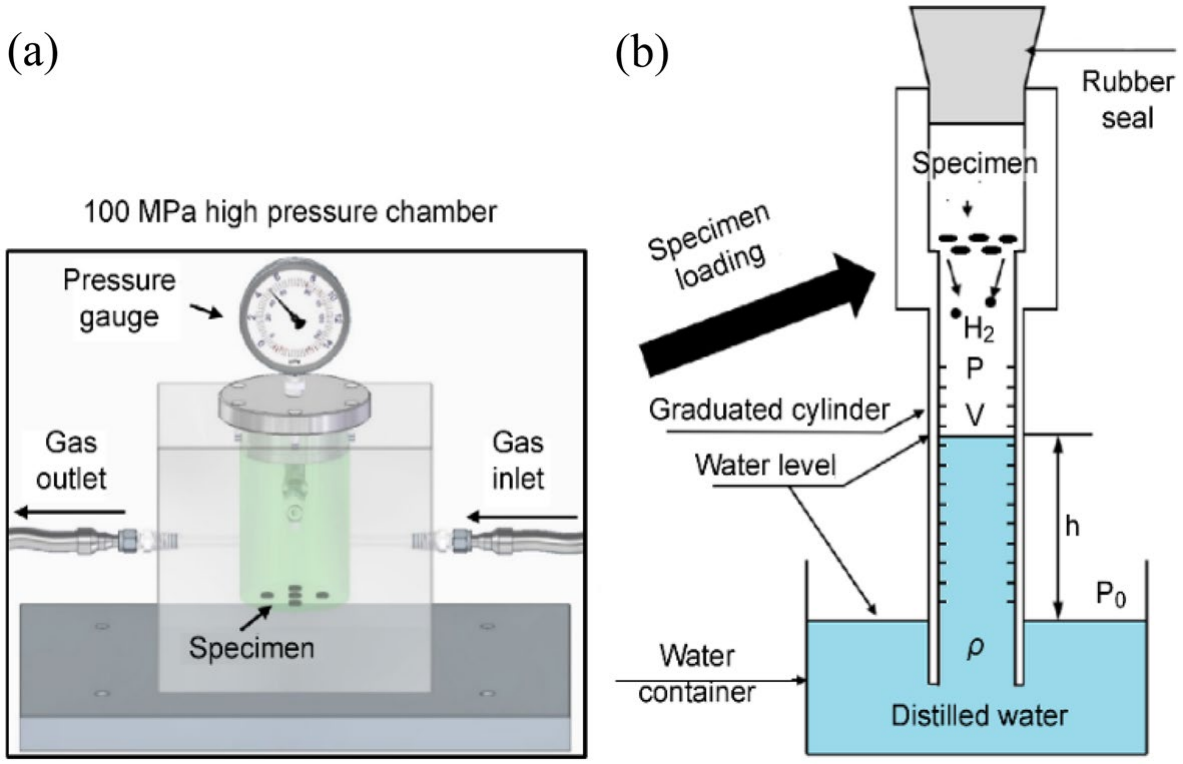
Illustration of the volumetric measurement system to measure the gas released by a specimen after exposure to HP gas and subsequent decompression. (**a**) Specimen exposed to gas in an HP chamber. (**b**) After decompression in the chamber, the specimen was loaded into a graduated cylinder. The cylinder was partially immersed in a container of water, and hydrogen emission measurements were conducted. Blue in graduated cylinder indicates distilled water.

In the previous section, we described the two sensing methods for measuring hydrogen leaks, diffusivity and permeability. The diffusivity was obtained using the volumetric system shown in Fig. 1. Thus, we applied Fick’s second diffusion equation. By assuming that hydrogen desorption followed the Fickian diffusion, the concentration ${{\text{C}}}_{{\rm{E}}}({\text{t}})$ of released H_2_ was expressed as^32,33^:

1 $$\begin{aligned}{\text{C}}_{{\text{E}}} \left( t \right)/{\text{C}}_{\infty } &= 1 -\frac{{32}}{{{\uppi}^{2} }} \times \left[ {\sum\limits_{{\text{n}} =0}^{\infty } \frac{\exp \left\{ \frac{{ - \left( {2n + 1}\right)^{2} {\uppi}^{2} {\text{Dt}}}}{{l^{2} }} \right\}}{\left({2{\text{n}} + 1} \right)^{2}}} \right] \times \left[{\sum\limits_{{\text{n}} = 1}^{\infty } \frac{\exp \left\{ { -\frac{{{\text{D}}{\upbeta}_{{\text{n}}}^{2} {\text{t}}}}{{\rho ^{2}}}} \right\}}{{\upbeta}_{{\text{n}}}^{2}}}\right] \\ &= 1 -\frac{32}{{\uppi}^{2}} \times \left[ {\frac{\exp \left( { -\frac{{{\uppi}^{2} {\text{Dt}}}}{{l^{2} }}} \right)}{1^{2}} +\frac{\exp \left( { - \frac{{{3}^{2} {\uppi}^{2}{\text{Dt}}}}{{l^{2} }}} \right)}{3^{2}} + \ldots , +\frac{\exp \left( { - \frac{\left( {{\text{2n}} + 1} \right)^{2}{\uppi}^{2} {\text{Dt}}}{l^{2}}} \right)}{({\text{2n}} +1)^{2}} + \ldots ,} \right] \\ &\quad \times \left[{\frac{{\text{exp}}\left( {{-}\frac{{{\text{D}}{\upbeta}_{{1}}^{2}{\text{t}}}}{{{\uprho}^{2} }}} \right)}{{\upbeta}_{{1}}^{2} }+\frac{{\text{exp}}\left( {{ { - }}\frac{{{\text{D}}{\upbeta}_{2}^{2} {\text{t}}}}{{{\uprho}^{2} }}}\right)}{{\upbeta}_{2}^{2}} + \ldots , +\frac{{\text{exp}}\left( {-\frac{{\text{D}}{\upbeta}_{{\text{n}}}^{2}{\text{t}}}{{\uprho}^{2}}} \right)}{{\upbeta}_{{\text{n}}}^{2}}+ \ldots ,} \right] \end{aligned}$$

where ${\upbeta }_{{\text{n}}}$ is the root of the zeroth-order Bessel function J_0_(β_n_) of β_1_ = 2.40483, β_2_ = 5.52008,…, β_50_ = 156.295. Equation (1) are infinite series expansions with two summations. The equation provided the solution for Fick’s second diffusion equation for a cylindrical specimen. In equation, C_E_ = 0 at *t* = 0 and C_E_ = ${{\text{C}}}_{\infty }$ at *t* = ∞; ${{\text{C}}}_{\infty }$ is the saturated total H_2_ concentration at infinite time, i.e., the H_2_ total uptake; D is the diffusivity; $\rho$ is the radius and $l$ is thickness (height) of the cylindrical sample.

Because Eq. (1) is complicated one with two infinite summations, we developed a diffusion analysis program that calculated D and ${{\text{C}}}_{\infty }$ using 100 terms in the first summation and 50 terms in the second summation $({\upbeta }_{50}$) in Eq. (1). By applying the diffusion analysis program, we analyzed the ${{\text{C}}}_{{\text{E}}}\left({\text{t}}\right)$ data using Eq. (1) and a simplex nonlinear optimization algorithm^34–36^.

**Figure 2:**
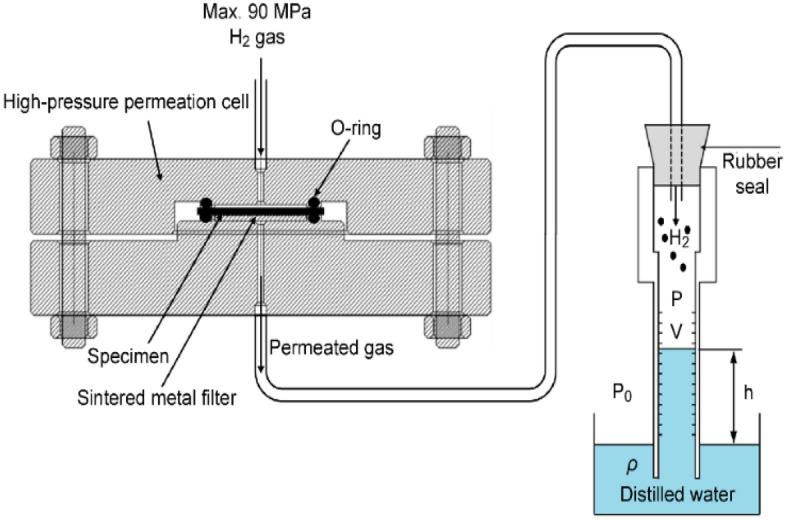
Volumetric analysis system for measuring hydrogen permeation under HP by using a permeation cell and graduated cylinder. The cylinder is the same as that shown in Fig. 1.

Similar to the measurement of the H_2_ concentration after decompression, as shown in Fig. 1, the diffusivity and permeability were obtained using the permeation cell shown in Fig. 2. We expressed the number of moles of diffusing gas per unit area (*A*) *Q*(*t*) as follows^32^:

2 $$Q\left( t \right) = \frac{{n\left( t \right)}}{A} = C_{1} \times \left[ {\frac{{Dt}}{l} - \frac{l}{6} - \frac{{2l}}{{\pi ^{2} }}\mathop \sum \limits_{1}^{\infty } \frac{{\left( { - 1} \right)^{n} }}{{n^{2} }}\exp \left( {{\raise0.7ex\hbox{${ - Dn^{2} \pi ^{2} t}$} \!\mathord{\left/ {\vphantom {{ - Dn^{2} \pi ^{2} t} {l^{2} }}}\right.\kern-\nulldelimiterspace} \!\lower0.7ex\hbox{${l^{2} }$}}} \right)} \right]$$

where C_0_ is the initial concentration of the polymer sheet for 0 < *x* < *l* at *t* = 0, C_1_ is the concentration at *x* = 0 in the HP part, and C_2_ is the concentration at *x* = *l* in the low-pressure part. According to the experimental arrangement in the permeation cell, for a specimen with thickness *l*, the polymer specimen was initially at zero concentration (C_0_ = 0), and the concentration at the face (*x* = *l*) through which the gas diffused was maintained effectively at zero concentration (C_2_ = 0).

The summation with the infinite series expansion in Eq. (2) included 50 terms in the actual calculation. Because the terms above the 50th term were sufficiently small and negligible, i.e., less than 10^–5^, we developed a dedicated diffusion–permeation analysis program to analyze 50 terms and obtain the diffusion coefficient by Eq. (2). This method was more precise than the time lag (*L*) method obtained by a simple approximated equation after reaching infinite time $L = l^{2} /6D$^32^.

The permeation was deduced as follows from the linear slope $(\frac{\Delta n}{\Delta t})$ of moles of hydrogen with respect to time:

3 $$P=\frac{(\frac{\Delta n}{\Delta t})l}{A\Delta P}$$

where *A* is the gas contact area of the specimen and $\Delta P$ is the pressure gradient between the feed side and the permeate side in the permeation cell. From the steady-state flow rate, the gas permeability was deduced by Eq. (3).

## Measurement procedure for obtaining the hydrogen uptake and diffusivity values

**Figure 3:**
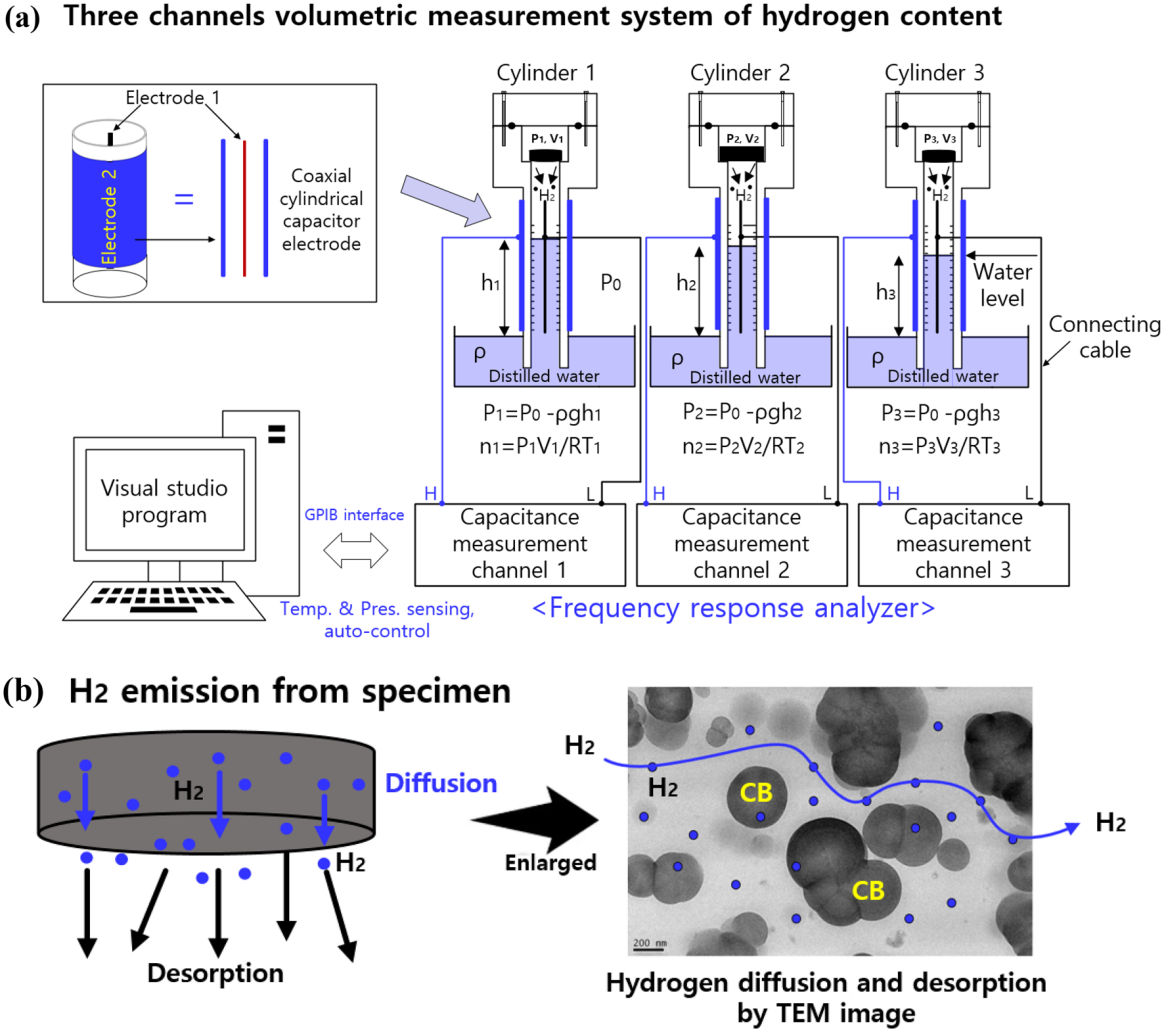
(**a**) System diagram of the three-channel volumetric measurement with three coaxial cylindrical capacitive electrodes in three cylinders and an FRA with a GPIB interfaced with a programmed PC. Blue in graduated cylinder indicates distilled water in the water containers and graduated cylinders. (**b**) H_2_ emissions from specimens inside the three cylinders. Left: H_2_ diffusion in the specimen and desorption outside the specimen, right: TEM image indicating the H_2_ diffusion path and desorption.

Figure 3a presents a three-channel volumetric analysis system with three coaxial cylindrical electrodes, three cylinders and an FRA with GPIB interfaced with a PC operating a visual studio program. The FRA for capacitance measurement used a VSP-300 impedance analyzer with a GPIB interface connected to a PC and an automated measurement program. The measurements covering from the low-frequency range to high frequency (from 0.01 Hz to 7 MHz) were performed at an applied voltage of 500 mV using VSP-300. The overall accuracy of the impedance analyzer is less than 1% according to the manufacturer specification, except for at low frequencies below 1 Hz. The impedance value at the same frequency was obtained by averaging after repeated measurements of 400 times at 1 MHz. This sensor system was developed in-house.

An automated measurement program operated by VSP-300 is developed by Microsoft Visual Studio 2019 for data collection. The program language was written using C#. In our developed program, we could set the parameters through the graphical user interface (GUI), such as number of channel, frequency, number of internal/external measurement, measurement time limit, measuring interval. For the data analysis, we used a diffusion analysis program developed using Visual Studio to calculate *D* ${\text{and}}$ ${C}_{\infty }$ based on least-squares regression.

Three specimens in Fig. 3a were simultaneously measured by the three-channel system. We could measure further samples by increasing the number of cylinders. A schematic diagram of the three-channel volumetric measurement system is shown in which three cylinders stood in containers filled with distilled water. Three FRA channels connected in parallel were employed for automatic real-time and simultaneous capacitance measurements of the gas–water mixtures in cylinders with three sets of coaxial cylindrical electrodes. Electrodes with different radii were used to evaluate the performance levels of the sensor. Figure 3b shows the H_2_ emissions from the specimens, with the H_2_ diffusion path shown by transmission electron microscopy (TEM).

**Figure 4:**
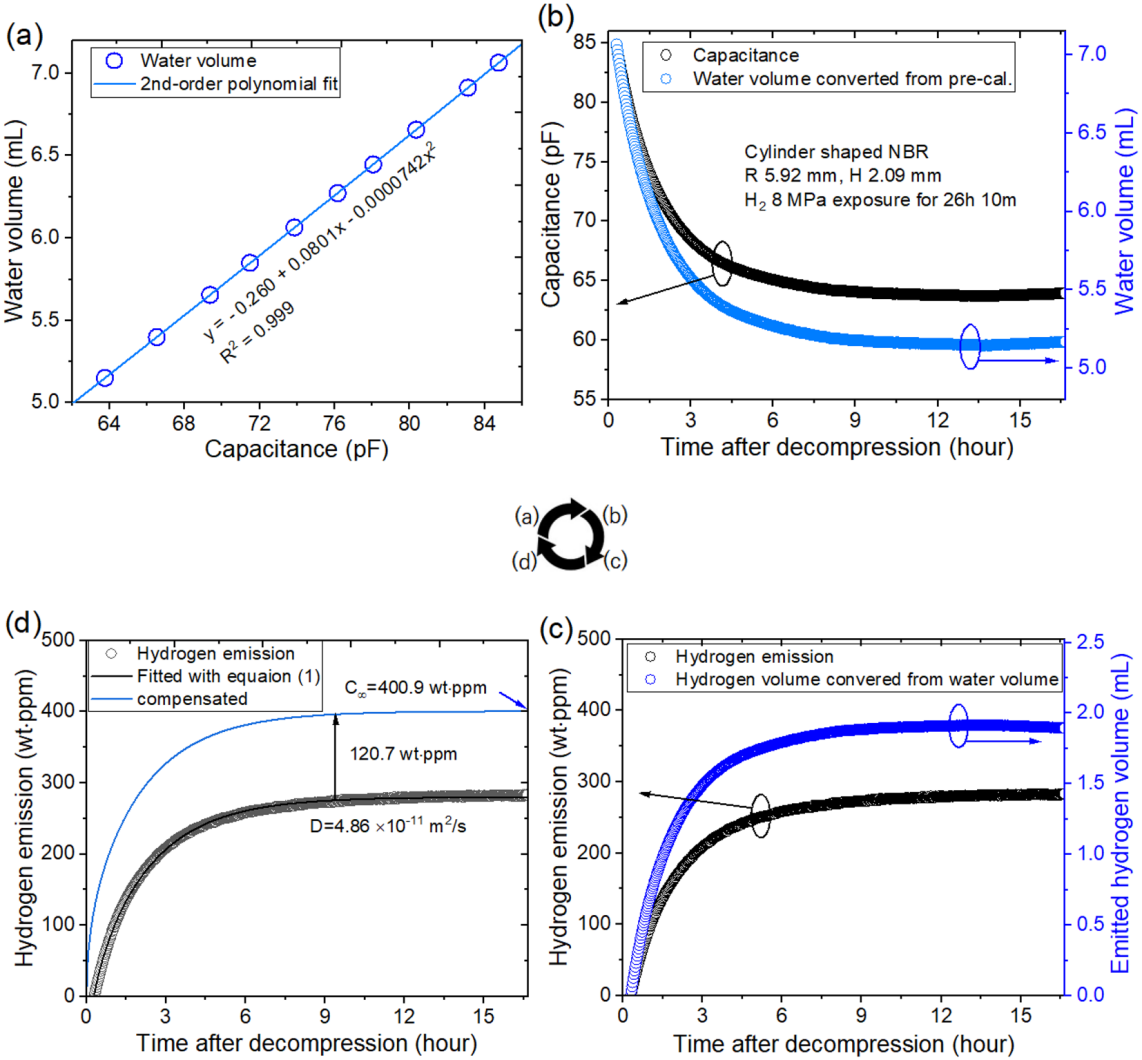
Overall sequence of the acquisition of the diffusion parameters for a cylindrical NBR polymer specimen by using coaxial cylinder capacitive electrodes and an FRA. (**a**) Pre-calibration result described as a second order polynomial equation between the water volume and capacitance by quadratic regression. (**b**) Time-dependent water level converted from the capacitance, with black and blue circles corresponding to the capacitance and water volume, respectively. (**c**) Water volume converted to emitted hydrogen gas volume and then to hydrogen emission in units of wt·ppm through Eq. (3). (**d**) Diffusivity,* D*$,\mathrm{and total uptake},$ ${C}_{\infty }$, determined using a diffusion analysis program and Eq. (5). The blue line in (**d**) is the total compensated emission curve obtained by restoring the missing content caused by the time lag.

The gas released by each specimen lowered the water level with increasing time. By a programmed capacitor measurements with coaxial cylindrical electrodes and a diffusion analysis program, the diffusion/hydrogen concentrations for the samples were determined. Figure 4a, b, c and d show the entire process for obtaining the hydrogen uptake and diffusion values in the cylindrical NBR polymer specimen:

(A) The process for obtaining the associated equation from precalibration data was as follows. We measured the water level against the capacitance at the corresponding channel; there was decreasing water level in the cylinder without a sample. The change in the capacitance due to the change in water volume was measured by an FRA with electrodes. The position of the water level was measured in units of pixels by a digital camera. Then, a 2nd-order polynomial equation for the correlation between the water volume and capacitance was obtained by quadratic regression with an excellent correlation coefficient of R^2^ = 0.999, as shown in Fig. 4a.
(B) According to the pre-calibration equation, the capacitance was converted to the water volume (Fig. 4b). The black and blue circles corresponded to the capacitance and water volume, respectively, and they were compared against the elapsed time after decompression.
(C) The water level was converted to the volume of emitted hydrogen gas, which was then converted to hydrogen emission in unit of wt·ppm through Eqs. (4)–(6) (Fig. 4c).
(D) The diffusivity and total uptake (*D* ${\text{and}}$ ${C}_{\infty }$) were determined by a diffusion analysis program by Eq. (1) based on the least-square fits. The final H_2_ content (400.9 wt·ppm) was obtained by compensating for the offset value (120.7 wt·ppm) that was missed due to the time lag (Fig. 4d).

## Performance testing and validation of the developed sensor system

We evaluated the performance of the gas sensing system by utilizing several approaches, including performance testing and validation of the sensor system.

### Measurement of both hydrogen leaks and hydrogen permeation

**Figure 5:**
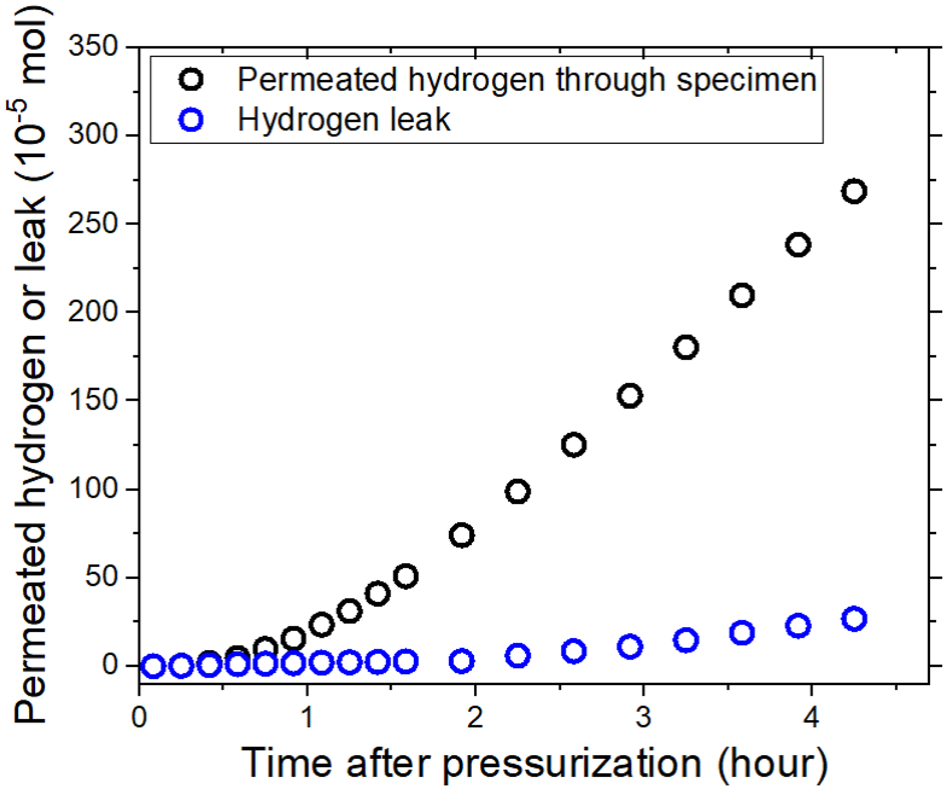
Permeated hydrogen and leaked hydrogen vs. time after pressurization of a cylindrical specimen of EPDM polymer.

By using volumetric analysis based on the permeation cell and coaxial capacitive electrode (Fig. 2), we monitored the permeation through the specimen and hydrogen leaks caused by fracture. The results provided in Fig. 5 reveal the real-time permeated hydrogen and leakage contents in units of moles of hydrogen versus time after pressurization for cylindrical EPDM specimens. The permeated hydrogen was measured through diffusion from the specimen after reaching a steady state. The small content from the leak caused by fracture in the specimen increased linearly with time.

**Figure 6:**
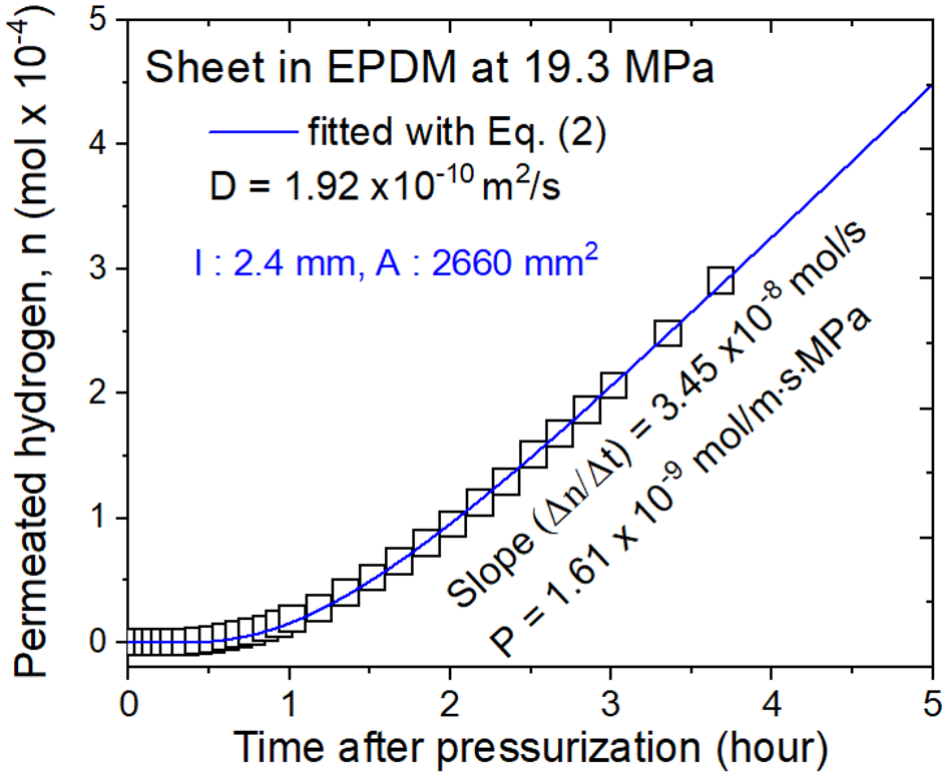
Permeation/diffusion measurements for a sheet of EPDM polymer. The slope of the blue line was obtained by the linear data after reaching steady state conditions for a specified number of moles of permeated hydrogen n with regard to time after pressurization.

Hydrogen leakage due to permeation through a polymer membrane was measured by the differential pressure method. In Fig. 6, we present the moles of permeated hydrogen vs. time after pressurization for the case of a sheet of EPDM used as a gas seal. The diffusion coefficient and permeation were obtained according to Eqs. (2) and (3), respectively, by applying the diffusion–permeation analysis program. The diffusion coefficient was accurately obtained by Eq. (2) instead of by the approximate time lag method. The gas permeability was obtained from the steady-state flow rate.

### Dual-mode and single-mode H_2_ diffusion monitoring in polymers

**Figure 7:**
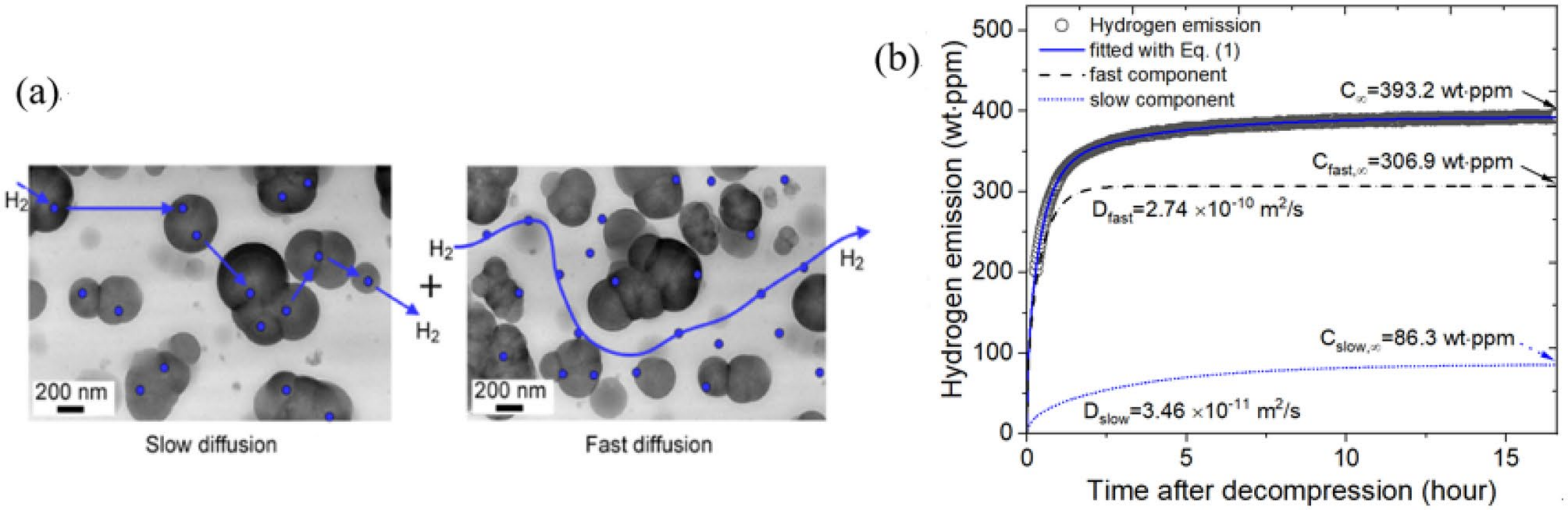
(**a**) Dual-mode H_2_ diffusion model in a cylindrical specimen of EPDM polymer. The black spheres indicate CB. The blue circles indicate H_2_. The blue line is the path of H_2_ diffusion. Left: slow diffusion of H_2_ adsorbed at the CB–filler interface. Right: fast diffusion of H_2_ absorbed in the polymer network. (**b**) Dual-mode emission vs. time in cylindrical EPDM specimens with R = 5.90 mm and H = 2.35 mm after exposure to 8 MPa H_2_ for 26 h. The offset value included in the hydrogen emission data, is the sum of the dotted blue and dashed black lines corresponding to slow and fast diffusion, respectively.

In previous investigations of the hydrogen diffusion mechanisms of EPDM composites blended with carbon black (CB)^37,38^, the hydrogen uptake and diffusion characteristics exhibited two types of diffusion: fast diffusion due to H_2_ absorbed in the polymer network and slow diffusion due to H_2_ adsorbed physically at the CB filler interface. The diffusion mechanism in CB-filled EPDM represented two diffusion behaviors, as shown in Fig. 7a, which were detected by the H_2_ gas sensor system developed in this work, as shown in Fig. 1. Figure 7b shows the H_2_ emission behavior vs. time data. The pressure depending sorption behaviors for pressures reaching 90 MPa were interpreted by the dual-mode model based on two contributions from Henry’s law and the Langmuir model^39,40^.

**Figure 8:**
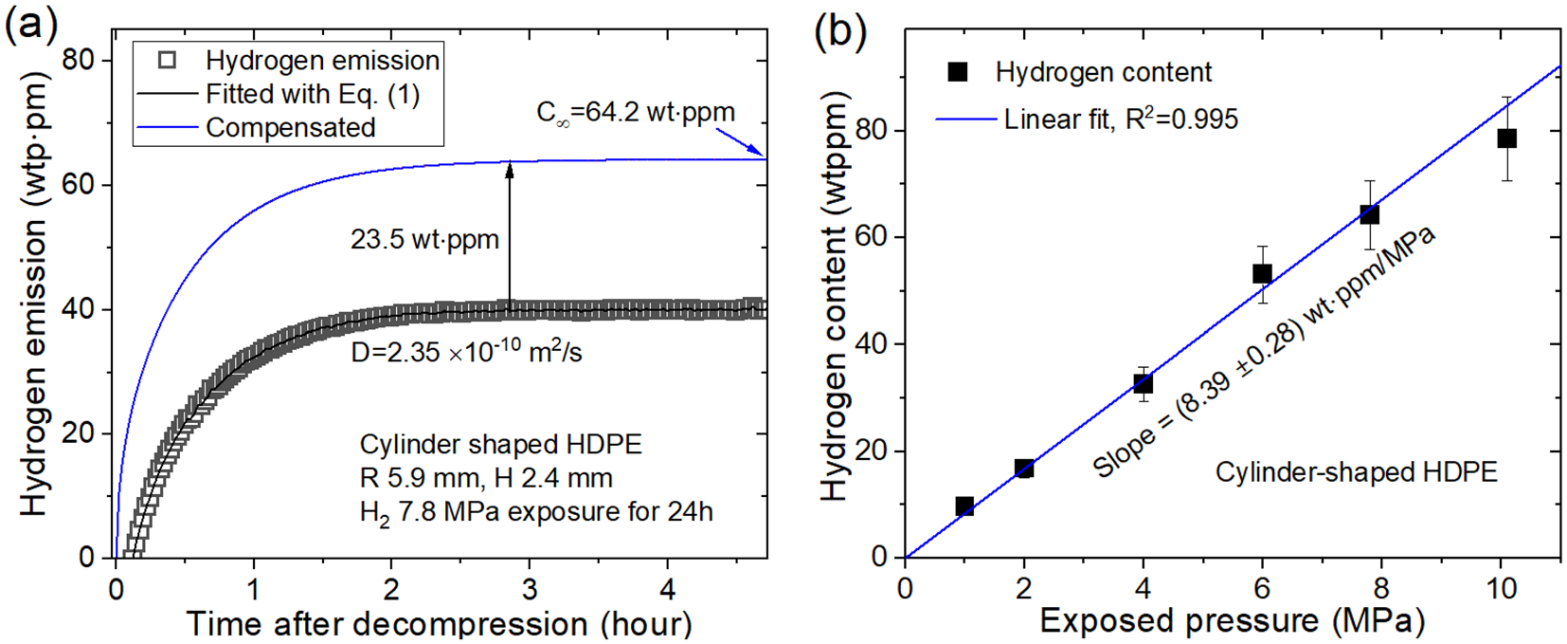
Single-mode H_2_ desorption/diffusion behavior in HDPE: (**a**) Hydrogen emission vs. time after decompression. The blue line is the compensated value obtained by adding the offset value (23.5 wt·ppm). (**b**) Linear relationship between hydrogen emission content and pressure following Henry’s law.

However, a single H_2_ diffusion behavior was observed in neat EPDM and HDPE plastic, which corresponded to fast diffusion in the polymer networks. The single-mode behavior for HDPE, as shown in Fig. 8a, was caused by fast H_2_ diffusion. The hydrogen sorption in Fig. 8b for the case of single diffusion/emission for HDPE linearly increased with increasing pressure, consistent with Henry’s law. The single and dual diffusion behaviors in Figs. 7 and 8 were demonstrated by the hydrogen gas sensing system based on a transparent coaxial capacitive electrode, as shown in Figs. 1 and 3.

The absorption and desorption processes of H_2_ were reversible in most cases, and they were ascribed to physisorption rather than chemisorption by introducing H_2_. These investigations were consistent with previous reports suggesting that H_2_ exposure did not change the chemical structure or chemical interactions in NBR according to nuclear magnetic resonance research^41,42^. These results were consistent with the analysis from a previous investigation^37,43^. Because of these investigations, the mechanism of hydrogen sensing could not be described with a series of related chemical equations.

### Other applications using sensor systems

**Figure 9:**
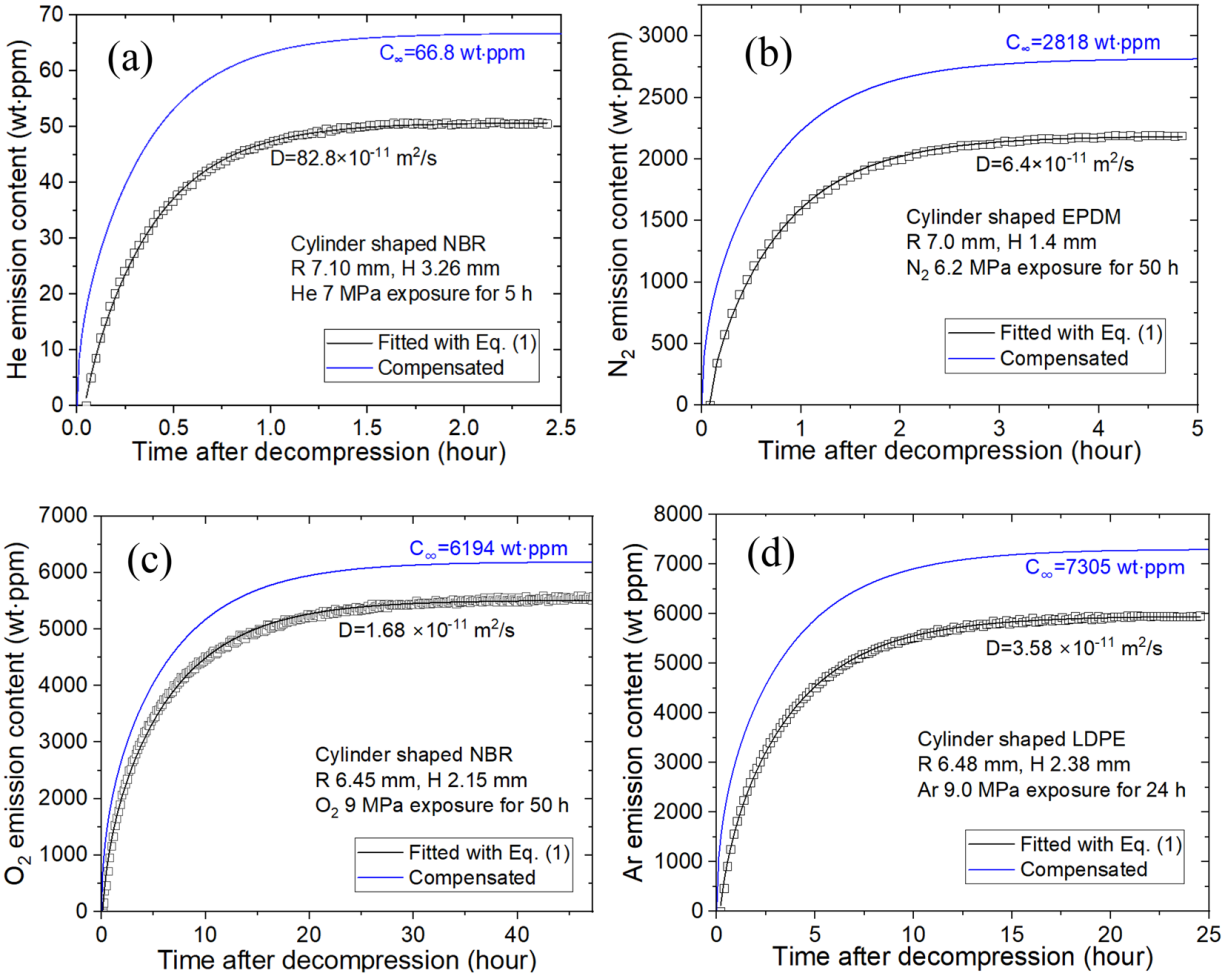
Gas uptake and diffusivity for (**a**) He, (**b**) N_2_, (**c**) O_2_ and (**d**) Ar obtained by the developed capacitive gas sensor in polymer specimens. H and R indicate the thickness and radius, respectively, of the cylindrical specimens.

The developed sensor system could be applied to other gases, such as He, N_2_, O_2_ and Ar. Similar to H_2_ sensors, gas sensors were utilized to detect the diffusivity and permeability values of other gases in polymers. The principle and process for sensing the gases were exactly the same irrespective of gas species with different masses per mole for the corresponding gas. For instance, the molar mass of N_2_ gas was 28.001 g/mol. We tested the corresponding gas uptake and diffusivity levels of He, N_2_, O_2_ and Ar (Fig. 9) for polymer specimens to demonstrate the sample applications.

Furthermore, the diffusion rate of gas in stainless steel was generally very slow at 10^–15^ m^2^/s at room temperature, and the gas sorption content in this material was smaller than that in polymer membranes. Thus, it was not easy to measure the permeation parameters in other materials. However, by using the developed sensor system with good sensitivity, the diffusivity of gas in thin sheets of metal with thicknesses of 0.1 mm could be measured and analyzed.

### H_2_ gas sensor performance tests

We tested various performance indicators of the sensor system with four kinds of coaxial capacitance electrodes with different *R*_1_ and *R*_2_ values. The performance indicators included the sensitivity, resolution, stability, measurable range, response time, figure of merit (FOM) and squared correlation coefficient (R^2^). The sensitivity of the sensor, defined as the change in the capacitance with regard to the change in water volume, was measured through the precalibration process described previously in Fig. 4a. The linear slope in Fig. 4a indicates the sensitivity of the capacitive sensor. The sensitivity of the four electrodes ranged from 9.2 to 21.9 pF/mL. The sensor of a high sensitivity indicates an improved resolution. The resolution implies the minimum measurable value of the water level (∆h = 0.01 cm) that corresponds to the minimum cylinder reading calculated from the pixel measurement by the digital camera. The resolution, which was determined as the mass concentration corresponding to a water level of 0.01 cm, ranged from 0.15 to 1.0 wt·ppm. However, the resolution could be reduced to less than 0.1 wt·ppm with increasing the sample number (or sample mass) or by using a graduated cylinder with a small inner radius R_2_.

Furthermore, the stability of the sensor system could be defined as the standard deviation obtained by measurement for 24 h after the measurement of hydrogen emission was completed, which amounted to 0.11 ~ 0.20%. The measurable range indicated the maximum allowable concentration per mass in cylinders with volume capacities of 10 mL, 20 mL and 50 mL. The measurable range could be adjusted by changing the sample mass and volume capacity of the cylinder. The response time in the gas sensor was associated with contributions from both water level sensing in graduated cylinder and capacitance measuring instrument including controlled measurement program. Thus, we may describe the response time from following two contributions;

- When the gas from specimen in graduated cylinder is emitted, the water level in graduated cylinder immediately decreased without any time delay according to an ideal equation of gas (PV = nRT).
- The FRA for capacitance measurement used a VSP-300 impedance analyzer with a GPIB interface connected to a PC and an automated measurement program using Visual Studio. The response time from capacitance measuring instrument controlled by automatic program was associated with the fast repetition frequency of 1 MHz and the number of measurements in one cycle in the FRA used for capacitance measurement. The time needed for the repeated measurements of 400 times at 1 MHz frequency using FRA is less than 0.1 s. In addition, the time resolution obtained finally from the entire system is 0.1 s. Thus, we estimated with a margin the response time did not exceed 1 s. The response time in Table 1 is estimated roughly to ∼1 s. Thus, the response time did not exceed 1 s.

**Table 1:** Performance test results for four sensing systems with different coaxial capacitor electrodes.

Performance	Sensor 1	Sensor 2	Sensor 3	Sensor 4
Specifications	R_1_ = 0.8 mm	R_1_ = 0.8 mm	R_1_ = 0.8 mm	R_1_ = 1.2 mm
	R_2_ = 5.0 mm	R_2_ = 4.0 mm	R_2_ = 2.5 mm	R_2_ = 2.5 mm
Sensitivity	9.2 pF/mL	11.7 pF/mL	18.8 pF/mL	21.9 pF/mL
Resolution	1.0 wt·ppm	0.59 wt·ppm	0.25 wt·ppm	0.15 wt·ppm
Stability	0.11%	0.14%	0.18%	0.20%
Measurable range	1500 wt·ppm	1365 wt·ppm	582 wt·ppm	477 wt·ppm
Response time	~ 1 s	~ 1 s	~ 1 s	~ 1 s
FOM	0.3%	0.4%	0.7%	1.0%
R^2^	0.999	0.999	0.999	0.995

The FOM for performance indicated the standard deviation between the measured data and the values calculated from Eqs. (1) and (2). FOM values less than 1% for the four sensor systems indicated a good consistency between the measured and theoretical values. R^2^ is the squared correlation coefficient between the capacitance and water volume determined from prec-alibration. The value in R^2^ (0.99) showed good correlation with linearity between capacitance and water volume. The performance results for the developed hydrogen capacitive sensor system are summarized in Table 1. In addition, we could adjust the measuring range, resolution, and sensitivity values because the specimen number, cylinder volume capacity and sensitivity were changeable. In summary, all performance tests indicated that the four sensors with different specifications were excellent for hydrogen gas sensing. However, we could select a suitable sensor depending on the experimental purpose.

### Validation by comparison with other sensor systems

**Figure 10:**
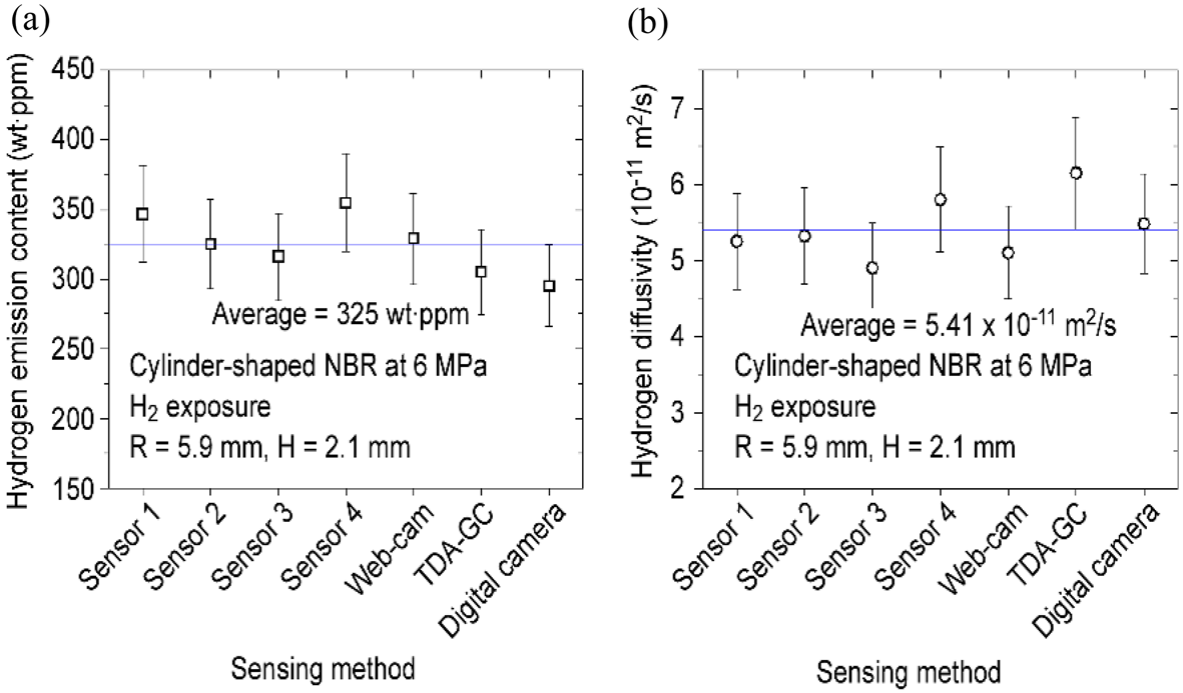
Comparison of various sensing methods for the (**a**) H_2_ emission content and (**b**) Diffusion coefficient. The four sensor systems indicate the capacitive electrodes with different radii presented in Table 1.

The developed H_2_ sensor system was validated by comparing its measured values with those measured by different methods. Thus, the hydrogen emission content and diffusivity measured for the same polymer specimen by the electrode capacitive sensor developed in this work were compared with those obtained by other methods. The results obtained by various sensors with different electrode radii were included for comparison. The other methods corresponded to volumetric analysis according to a webcam analysis^44^, thermal desorption analysis–gas chromatography analysis^38^, and digital camera analysis^36^, which were verified in previous studies^36,38,44,45^. The comparison results for various sensing methods were consistent with each other within the expanded uncertainty level, as shown in Fig. 10.

## Conclusions

Hydrogen gas sensors could play significant roles in retaining safety and protecting property where H_2_ was produced, transported, used and stored. Thus, we developed a H_2_ sensor system based on volumetric measurements using a graduated cylinder with coaxial capacitive electrodes and transparent conductive film. The capacitance changes in the electrodes were related to the changes in water volume due to released hydrogen, resulting in accurate measurements of hydrogen content. By applying a diffusion–permeation analysis program, the developed capacitive hydrogen sensor could detect hydrogen leaks and concentrations with the diffusivity due to permeation from the specimens. The sensor system consisted of a high-pressure chamber, a volumetric analysis system with a graduated cylinder, a permeation cell with a graduated cylinder, coaxial capacitor electrodes and a diffusion–permeation analysis program to measure the hydrogen gas leakage caused by imperfect connecting parts and the concentration, diffusivity, and permeation values of H_2_ emitted from specimens enriched by H_2_ under high-pressure conditions.

The H_2_ sensor system exhibited multiple characteristics: a low detection limit of 0.15 wt·ppm H_2_ content, a measurable range of 1500 wt·ppm, a stability of 0.11%, and a fast response time of one second. This remarkable performance was enabled by precalibration with automatic capacitance measurements by using a frequency response analyzer, which removed noise by averaging several hundred repetitive measurements with a fast 1 MHz repetition rate. In addition, the insensitivity of the sensor to variations in temperature/pressure enabled its application for gas detection and the characterization of pure gases (He, N_2_, O_2_ and Ar) through real time measurements.

The features of the sensor techniques and applications developed in this study are summarized as follows:

- This technique could simply evaluate the permeation and diffusion of gas through polymer materials enriched with gas under high-pressure conditions.
- This technique was insensitive to gas species and sample shape.
- A technique for precise calculations was developed by a diffusion–permeation analysis program.
- This technique had an adjustable range, sensitivity and resolution for measurements.
- This technique was visual because the processes of gas emission and leaks could be observed by the change of water level.
- This technique was independent because it did not involve chemical reactions between the target gas and specimen.

The developed gas sensor can be utilized in the testing of permeation properties, such as leakage and sealing ability of rubber material and O-ring under high pressure for hydrogen fueling stations, gas industry and rubber seal production company. However, for use of real-life hydrogen sensing, the sensing system should be minimized and is portable by using compact capacitance meter and digital camera instead of massive frequency response analyzer.

## Methods

### Sample preparation and high-pressure gas exposure

We used NBR and EPDM polymer specimens as O-ring components in H_2_ infrastructure systems. Additionally, HDPE samples were used as a liner material for type IV pressure vessels of fuel cell electric vehicle; these liners were included in the assessment of hydrogen sensor performance. The composition and density of the polymer samples were provided in literatures^38,46^. The shapes and dimensions of specimens are as:

- Cylindrical NBR specimens with radii of 5.9 mm and thicknesses of 2.1 mm
- Cylindrical EPDM specimens with radii of 5.9 mm and thicknesses of 2.3 mm

HDPE fabricated with antimicrobial technology was utilized in the volumetric experiment. The physical property of the HDPE samples were described previously in literature^44^. HDPE specimens with the following shapes/dimensions were prepared:

- Cylindrical HDPE specimens with radii of 5.9 mm and thicknesses of 2.4 mm
- Cylindrical LDPE specimens with radii of 6.5 mm and thicknesses of 2.4 mm

A stainless steel chamber was used to expose specimens to gas at specified HPs and a temperature of 298 K prior to volumetric measurement. Conducting H_2_ exposure for 24 h was sufficient to attain an equilibrium state for gas sorption. After exposure to HP gas, the valve was opened and the gas in the HP chamber was emitted. After the decompression, the elapsed time was counted from the time, *t* = 0, which the gas pressure inside HP chamber reached atmospheric pressure. Moreover, the H_2_ permeation was measured by employing the differential pressure method at the desired constant pressure and a temperature of 298 K on the HP side of the permeation cell. The purities of pure gases in this study were as. H_2_: 99.99%, He: 99.99%, N_2_: 99.99%, O_2_: 99.99% and Ar: 99.99%.

### Volumetric measurements for H_2_ concentration

We established two different H_2_ exposure techniques based on volumetric systems to measure hydrogen diffusion/permeation. One method was the measurement of the hydrogen concentration released from the specimen after exposure to HP and after subsequent decompression^38,44,46^. Figure 1 shows a volumetric analysis system to measure the content of released H_2_ at a temperature of 298 K, which consisted of an HP chamber for H_2_ exposure and a graduated cylinder that was immersed in a water container.

After exposure to gas in the HP chamber and after decompression, a specimen was loaded in the air volume at the upper side in a cylinder, as shown in Fig. 1. The H_2_ gradually emitted from the specimen lowered the water level in the graduated cylinder after decompression. Therefore, the pressure ($$P)$$ and volume ($$V)$$ of the gas inside the graduated cylinder changed over time.

The gas in the graduated cylinder followed the ideal equation of gas (*PV* = *nRT*), where $R$ is the gas constant 8.20544 × 10^–5^ m^3^·atm/(mol·K), *T* is the gas temperature in the graduated cylinder, and *n* is the mole number of H_2_ gas released in the graduated cylinder. The time dependences of $P(t)$ and $V(t)$ of the gas in the cylinder were written as^38,44,46^:

4 $$P\left( t \right) = P_{o} \left( t \right) - \rho gh\left( t \right),\;V\left( t \right) = { }V_{o} - V_{s} - V_{h} \left( t \right)$$

${\mathrm{where }P}_{o}$ is the pressure at outside the graduated cylinder, *g* is the gravitation acceleration, $\uprho$ is the density of distilled water, $h(t)$ is the water level (height) in the graduated cylinder, ${V}_{o}$ is the total volume of gas and water in the graduated cylinder measured from the water level in the water container, ${V}_{h}(t)$ is the time-dependent volume of water in the cylinder and ${V}_{s}$ is the sample volume.

The amount of H_2_ gas emitted by the polymeric specimen was measured by reading the water position $\left[{V}_{h}\left(t\right)\right]$ against time. Thus, the total mole number [$n(t$)] of emitted gas was determined by measuring the total gas volume [$V(t)]$ in the graduated cylinder, i.e., the reduction in the water level, as follows:

5 $$\begin{aligned} n\left( t \right) & = \frac{P\left( t \right)V\left( t \right)}{{RT\left( t \right)}} = \frac{{P\left( t \right)\left[ {V_{A} + V_{H} \left( t \right)} \right]}}{RT\left( t \right)}{ = }\frac{{P_{0} \left[ {1 + \beta \left( t \right)} \right]\left[ {V_{A} + V_{H} \left( t \right)} \right]}}{{RT_{0} \left[ {1 + \alpha \left( t \right)} \right]}} \\ & \cong \frac{{P_{0} }}{{RT_{0} }}\left[ {V_{A} + V_{H} \left( t \right) + V\left( t \right)\left( {\beta \left( t \right) - \alpha \left( t \right)} \right)} \right] = n_{A} \left( t \right) + n_{H} \left( t \right),{ } \\ \end{aligned}$$

$${\mathrm{with\, }n}_{A}\left(t\right)=\frac{{P}_{0}}{R{T}_{0}}{V}_{A}$$,$${n}_{H}\left(t\right)=\frac{{P}_{0}}{R{T}_{0}}\left[{V}_{H}(t)+V\left(t\right)\left(\beta (t)-\alpha (t)\right)\right]$$

$$\mathrm{\alpha }(t)=\frac{T(t)-{T}_{0}}{{T}_{0}}$$, $$\upbeta (t)=\frac{P(t)-{P}_{0}}{{P}_{0}}$$, where $${T}_{0}$$ and $${P}_{0}$$ are the initial temperature and pressure of the gas in the graduated cylinder, respectively, $$V(t)$$ is the sum of the remaining initial air volume ($${V}_{A})$$ and the released hydrogen volume $${[V}_{H}\left(t\right)]$$; i.e., $$V\left(t\right)={V}_{A}+{V}_{H}\left(t\right)$$, $${n}_{A}$$ is the initial mole number of air, and $${n}_{H}(t)$$ is the time-dependent mole number of hydrogen corresponding to the hydrogen volume increase resulting from the released hydrogen. Thus, $${n}_{H}(t)$$ was converted to the concentration of H_2_ [$$C\left(t\right)]$$ emitted per unit mass from the specimen as:

6 $$\begin{aligned} { }C\left( {\text{t}} \right)\left[ {{\text{wt}} \cdot {\text{ppm}}} \right] = & n_{H} \left( t \right)\left[ {{\text{mol}}} \right] \times { }\frac{{m_{H2} { }\left[ {\frac{{\text{g}}}{{{\text{mol}}}}} \right]}}{{m_{sample} \left[ {\text{g}} \right]}} \times 10^{6} \\ & = \frac{{P_{0} }}{{RT_{0} }}\left[ {V_{H} \left( t \right) + V\left( t \right)\left( {\beta \left( t \right) - \alpha \left( t \right)} \right)} \right]\left[ {{\text{mol}}} \right] \times \frac{{m_{H2} \left[ {\frac{{\text{g}}}{{{\text{mol}}}}} \right]}}{{m_{sample} \left[ {\text{g}} \right]}} \times 10^{6} \\ \end{aligned}$$

where ${m}_{H2}$[*g*/mol] is the molar mass of H_2_ gas 2.016 g/mol and ${m}_{sample}$ is the mass of the sample. According to Eqs. (5) and (6), the time-dependent mole number of H_2_ ${n}_{H}(t)$ was converted to the H_2_ mass concentration [$C\left(t\right)]$ by scaling with the factor $k=\left[ \frac{{m}_{H2} }{{m}_{sample}}\right].$ ${n}_{H}(t)$ and $C\left(t\right)$ were influenced by the fluctuations of temperature and pressure in the laboratory environment. To measure precisely, the variations due to changes in temperature and pressure could be compensated by automatic program according to Eq. (6). That is., the terms, $V\left(t\right)\left(\beta (t)-\alpha (t)\right),$ indicates the volume change in the ${V}_{H}\left(t\right),$ caused by the temperature and pressure variations. The compensation indicates the application of $V\left(t\right)\left(\beta (t)-\alpha (t)\right)$ calculation in Eq. (6). The application is automatically conducted by program with temperature and pressure recorded. Thus the insensitivity of the sensor to variations in temperature/pressure indicates application of automatic compensation by program.

The change in the water level due to the release of H_2_ was monitored by the capacitance of coaxial cylinder capacitor electrodes attached to the graduated cylinder. Moreover, the other H_2_ sensing system for obtaining the hydrogen leak, diffusivity, and permeability values was based on the differential pressure method^47^, which is called the time lag method under HP conditions; the method involved a permeation cell and a volumetric analysis technique, as shown in Fig. 2. The permeation cell was composed of a feed side under HP conditions and a permeated side under low-pressure conditions that were bordered by a polymer specimen that was serially connected through a stainless steel tube to a graduated cylinder to measure permeated H_2_. The change in water volume in the cylinder due to permeated H_2_ was monitored by the capacitance change in the electrode at 298 K. The principle and method used to obtain the number of moles of permeated H_2_ [$n\left(t\right)]$ from the water volume were the same as those given in Eqs. (5) and (6) and Fig. 1. The change in the water level due to permeated hydrogen was monitored by the capacitance of coaxial cylinder capacitor electrodes attached to the cylinder.

### Fabrication of coaxial cylindrical capacitive electrodes

**Figure 11:**
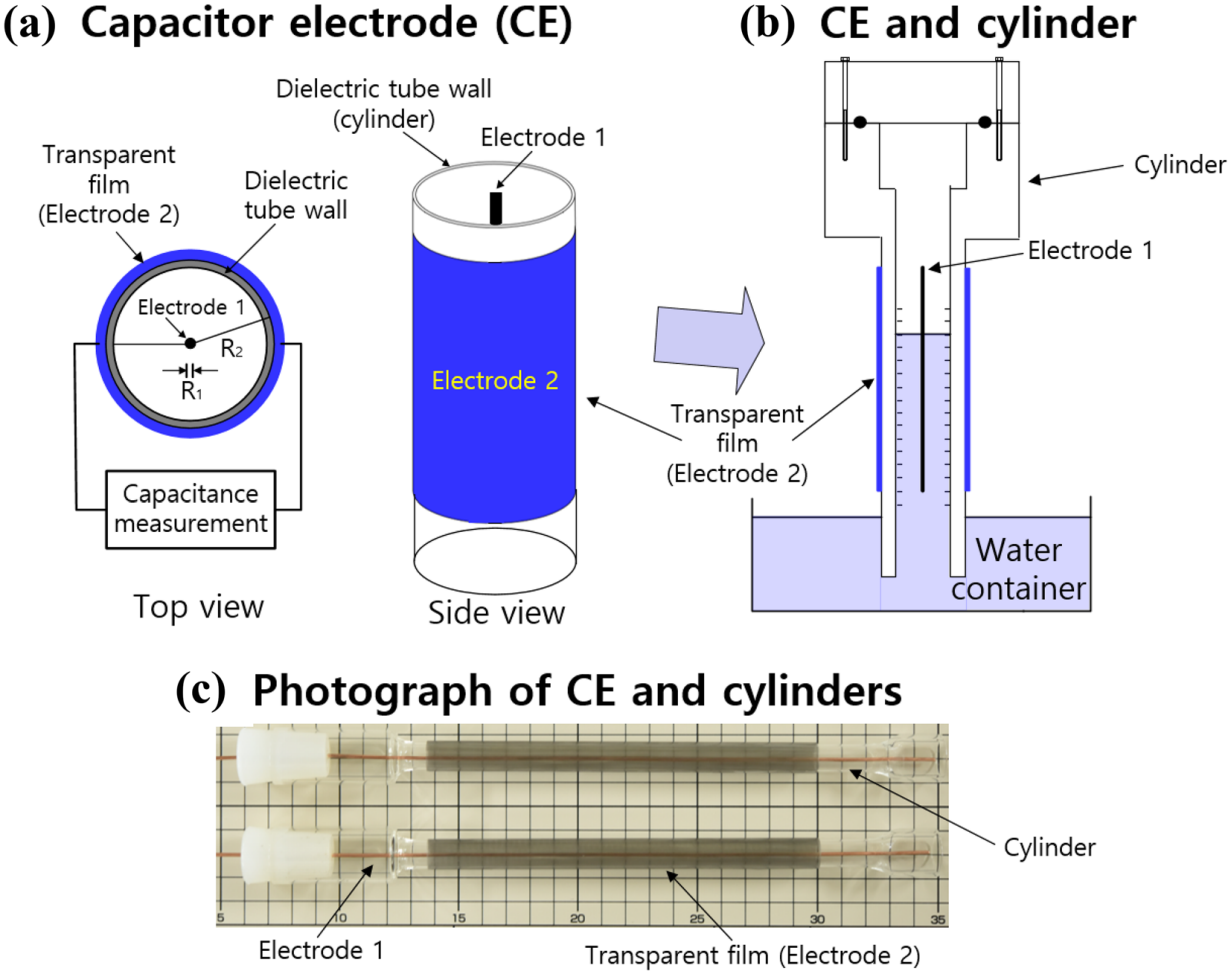
(**a**) Schematic structure of the coaxial capacitor electrode consisting of electrode 1 and electrode 2. (**b**) CE locations on the graduated cylinder immersed partially in the water container. (**c**) Photograph of electrode 1 and the transparent ITO thin film (electrode 2) attached to the external side of the cylinder. For clarity, transparent electrode 2 is indicated in blue.

To measure the capacitance change caused by the change in water volume in the cylinder, we used capacitive electrodes. Thus, the capacitive sensor was designed with coaxial cylindrical electrodes (electrodes 1 and 2) mounted at the center and outside faces of a cylinder; the cylinder was an acrylic tube (dielectric tube wall), as shown in Fig. 11a. A solid cylindrical conductor made of thin copper wire was used as electrode 1. A transparent thin film made of indium tin oxide (ITO), which was attached to the outside the cylinder, was used as electrode 2. A water–gas mixture filled the space between the two coaxial electrodes in the cylinder (Fig. 11b), where the blue and empty white spaces in the cylinder were occupied by water and gas, respectively. Photographs of the capacitor electrode (CE) and graduated cylinder are shown in Fig. 11c. The dielectric constant of water was 78.4 at 298 K^48^, which was larger than that of gas in a cylinder at 298 K. Thus, by using coaxial capacitive electrodes with good sensitivity, we detected the change in the water–gas volume that changed capacitance. The capacitance change $\Delta C$ with regard to the water level in the cylinder filled with a water–gas mixture was expressed by the following equations ^49^:

7 $$\Delta C = \frac{{2\pi \varepsilon_{0} \left( {\varepsilon_{w} h + \varepsilon_{g} \left( {L - h} \right)} \right)}}{{{\text{ln}}\left( {\frac{{R_{2} }}{{R_{1} }}} \right)}} = \frac{{2\pi \varepsilon_{0} \left( {\varepsilon_{w} - \varepsilon_{g} } \right)h}}{{{\text{ln}}\left( {\frac{{R_{2} }}{{R_{1} }}} \right)}} + \frac{{2\pi \varepsilon_{0} \varepsilon_{g} L}}{{{\text{ln}}\left( {\frac{{R_{2} }}{{R_{1} }}} \right)}}$$

where *L* is the axial length of the cylindrical capacitor, *R*_1_ is the inner radius of electrode 1, *R*_2_ is the inner radius of the coaxial cylinder shell (electrode 2), and ${\varepsilon }_{0, } {\varepsilon }_{{\text{w}}}$ and ${\varepsilon }_{{\text{g}}}$ are the permittivities of free space, water and gas, respectively.

For a fixed configuration with a coaxial cylindrical electrode, the second term on the right side of Eq. (7) was constant. Thus, $\Delta C$ was linearly proportional to the change in the water level, *h*. Thus, we determined the water level corresponding to the capacitance change with a pre-calibration equation of the relationship between the water level and the measured capacitance. From the water volume, we obtained the H_2_ concentration $C\left(t\right)$ according to Eqs. (5) and (6).

We fabricated four coaxial capacitance electrodes with different R_1_ and R_2_ values to discern any difference in their performance levels regarding sensitivity and stability. The results of the performance test were already presented in Table 1.

The sensor used in previous study is semi-cylindrical capacitance sensor^36^. The sensor fabricated with two semi-cylindrical electrodes mounted outside of an acrylic tube. An acrylic tube surrounded by two semi-cylindrical electrodes is filled with water–gas. The electrode attached to the outer wall of the acrylic tube is made of copper cylinder with a thickness of 1 mm.

Regarding comparison between two sensors, the hydrogen gas sensor by employing coaxial cylinder capacitive electrodes used in this work is superior to semi-cylindrical capacitance sensor, in views of sensitivity and resolution. The sensitivity and resolution in coaxial cylindrical capacitance sensor are ten times better than those in semi-cylindrical capacitance sensor. Thus this is reason why we have employed coaxial cylinder capacitance sensor in this study. However, the measurable range, stability and response time are almost similar with each other.

## Data Availability

The data used to support the findings of this study are available from the corresponding author upon request.
